# Immune dysregulation due to bi-allelic mutation of the actin remodeling protein DIAPH1

**DOI:** 10.3389/fimmu.2024.1406781

**Published:** 2024-07-15

**Authors:** Sagar Bhattad, Somashekara H. Ramakrishna, Ratan Kumar, Joseph M. Choi, Janet G. Markle

**Affiliations:** ^1^ Division of Pediatric Immunology and Rheumatology, Department of Pediatrics, Aster CMI Hospital, Bengaluru, India; ^2^ Department of Pediatric Hepatology, Gleneagles Health City, Chennai, India; ^3^ Department of Pediatrics, Tata Main Hospital, Jamshedpur, India; ^4^ Division of Molecular Pathogenesis, Department of Pathology Microbiology and Immunology, Vanderbilt University Medical Center, Nashville, TN, United States; ^5^ Division of Genetic Medicine, Department of Medicine, Vanderbilt Genetics Institute, Vanderbilt University Medical Center, Nashville, TN, United States; ^6^ Vanderbilt Center for Immunobiology, Vanderbilt Institute for Infection, Immunology and Inflammation, Nashville, TN, United States

**Keywords:** pediatrics, medical genetics, enteropathy, inborn errors of immunity, inflammatory bowel disease

## Abstract

Children with severe inflammatory diseases are challenging to diagnose and treat, and the etiology of disease often remains unexplained. Here we present DIAPH1 deficiency as an unexpected genetic finding in a child with fatal inflammatory bowel disease who also displayed complex neurological and developmental phenotypes. Bi-allelic mutations of *DIAPH1* were first described in patients with a severe neurological phenotype including microcephaly, intellectual disability, seizures, and blindness. Recent findings have expanded the clinical phenotype of DIAPH1 deficiency to include severe susceptibility to infections, placing this monogenic disease amongst the etiologies of inborn errors of immunity. Immune phenotypes in DIAPH1 deficiency are largely driven aberrant lymphocyte activation, particularly the failure to form an effective immune synapse in T cells. We present the case of a child with a novel homozygous deletion in *DIAPH1*, leading to a premature truncation in the Lasso domain of the protein. Unlike other cases of DIAPH1 deficiency, this patient did not have seizures or lung infections. Her major immune-related clinical symptoms were inflammation and enteropathy, diarrhea and failure to thrive. This patient did not show T or B cell lymphopenia but did have dramatically reduced naïve CD4+ and CD8+ T cells, expanded CD4-CD8- T cells, and elevated IgE. Similar to other cases of DIAPH1 deficiency, this patient had non-hematological phenotypes including microcephaly, developmental delay, and impaired vision. This patient’s symptSoms of immune dysregulation were not successfully controlled and were ultimately fatal. This case expands the clinical spectrum of DIAPH1 deficiency and reveals that autoimmune or inflammatory enteropathy may be the most prominent immunological manifestation of disease.

## Introduction

The protein DIAPH1 (Diaphanous homolog 1, also called mDia1) is one of 15 human formins (1, 2). Formins are highly conserved proteins that play a role in cytoskeletal remodeling by promoting the assembly and elongation of actin filaments via their conserved Formin Homology 2 (FH2) domains (1, 2).The first report of human autosomal recessive DIAPH1 deficiency was in 2015 (3). Studying a single consanguineous kindred, the authors used a combination of genome-wide linkage analysis and whole exome sequencing (WES) to identify five children homozygous for the *DIAPH1* mutation Q778X who had undetectable DIAPH1 protein (3). These children were affected with microcephaly, intellectual disability, seizures, short stature, and blindness. Although infectious or immunological phenotypes were not highlighted in this initial report of DIAPH1 deficiency, one of the patients died at age 18 of a chest infection (causal pathogen not reported), and another patient had a history of bronchiectasis (3). Shortly thereafter, another paper described bi-allelic *DIAPH1* mutations in four affected individuals from two unrelated consanguineous families (4). The affected children in these families had homozygous *DIAPH1* mutations F923fs/F923fs and R1049X/R1049X, respectively, and were diagnosed with postnatal microcephaly, early-onset epilepsy, severe visual impairment, and pulmonary symptoms including bronchiectasis and recurrent respiratory infections (4). In the single patient homozygous for the F923fs/F923fs mutation, these recurrent infections required admission to the intensive care unit (4). Among the three children with the R1049X/R1049X mutation, one died at age 13 of pneumonia (4). These findings suggested an important role for DIAPH1 in immunity, and particularly in protection from severe lung infections.

Recently, the role of DIAPH1 in T cell biology has been explored. Formins, and in particular DIAPH1, are highly expressed in T cells and are essential for Zap70-mediated phosphorylation of LAT following TCR stimulation (5). Upon TCR ligation by anti-CD3/anti-CD28, Zap70 is phosphorylated and Zap70 in turn phosphorylates LAT. However, when T cells were treated with formin-inhibiting drugs, Zap70, but not LAT, was phosphorylated in response to anti-CD3/anti-CD28 (5). High-resolution imaging revealed that localization of phosphorylated Zap70 to the immune synapse (IS) and subsequent LAT phosphorylation are critically dependent on formin-mediated actin polymerization (5). Due to its role in TCR-dependent LAT phosphorylation, the immunological aspects of DIAPH1 deficiency may phenocopy (completely or partially) those of LAT-deficient patients. Patients with LAT deficiency are affected by varied symptoms of immune deficiency and dysregulation, including recurrent infections and severe autoimmunity with infantile onset (6, 7). LAT deficiency is also associated with abnormal lymphocyte frequency and function, including progressive lymphopenia, reduced CD4+ and CD8+ T cell numbers, expansion of CD4-CD8- double negative T cells, and reduced activation and proliferation of T cells following anti-CD3/anti-CD28 stimulation (6, 7). These data suggested that aside from its critical roles in neurological development, human DIAPH1 was also indispensable for T cell-mediated immune responses.

Accordingly, a recent publication reported additional patients with bi-allelic *DIAPH1* mutations, and these patients displayed symptoms of combined immunodeficiency (CID) similar to those reported in LAT deficiency, in addition to microcephaly, seizures, cortical blindness, and developmental delay (8). Five Finnish patients were homozygous for the *DIAPH1* splice-donor variant c.684 + 1G>A despite no known shared recent ancestry (8). These patients had recurrent and severe infections suggestive of poor T cell responses, such as persistent *Molluscum contagiosum*, candidiasis, recurrent oral herpes lesions, and EBV viremia (8). One patient also had persistent vaccine strain Rubella skin infection and multifocal leukoencephalopathy positive for JC virus (8). Several of these patients had low T cell counts, and especially low CD4+ T cells, with CD4/CD8 ratios <1 (8). Patients had very low naïve CD4+ and CD8+ T cells (8). In agreement with the importance of formin-mediated actin polymerization at the immune synapse, T cells from DIAPH1-deficient patients did not properly position their microtubule-organizing center (MTOC) at the immunological synapse following T cell stimulation (8), and patients’ T cells had defective induction of activation markers CD69 and CD25, and poor proliferation in response to stimulation with anti-CD3/CD28 coated beads (8). These recent data highlight the importance of DIAPH1 to induction of cell-mediated immunity and define DIAPH1 deficiency as a monogenic disease affecting both neurodevelopment and lymphocyte function. We report the case of an infant presenting with inflammatory enteropathy, without any initial infections and without seizures, in whom we found a novel bi-allelic *DIAPH1* mutation.

## Methods

### Patients and healthy controls

This research study was approved by the Vanderbilt University Medical Center Institutional Review Board (IRB). Written informed consent was obtained for adult participants and parental consent was obtained for the patient.

### Whole exome sequencing

Genomic DNA was prepared from blood samples of the patient and her parents and used for trio-design whole exome sequencing (WES). Exome capture was performed using the Comprehensive Exome kit (Twist Biosciences). Paired-end sequencing was performed on a NovaSeq (Illumina) generating 150-base reads. WES data quality control was performed, data was aligned to the reference genome GRCH38/hg38, and variant calling was done according to Genome Analysis Toolkit (GATK) best practices and the BWA-MEM (9) alignment algorithm. Next, we used GATK (10)/Picard tools to sort the resulting files, performed base quality score recalibration, then used HaplotypeCaller (10) to call variants. Variant annotation was done using ANNOVAR (11). Variants were then filtered, to retain only variant with minor allele frequency <0.01 in the gnomAD (12) database, and which fit the autosomal recessive inheritance pattern for this family.

### Preparation of PBMC and Western blotting

Blood samples were diluted 1:1 with PBS + 2% FCS then added to SepMate tubes (Stemcell Technologies 85450) containing 15 mL Ficoll-Paque (Cytiva 17144002). The samples were then centrifugated at 1200 x g for 10 minutes at room temperature, and peripheral blood mononuclear cells (PBMC) were collected. PBMC were lysed in 25mM Tris, 0.15M NaCl, 1mM EDTA, 1% N40, 5% glycerol; pH 7.4 and protease inhibitor cocktail (Thermo Fisher A32953) on ice for 5 min. Lysate was cleared by centrifugation at 16,000 x g for 10 min then 30ug of cleared lysate from each of three healthy controls (HC1-HC3) and the patient were used for immunoblotting. Primary antibodies used were anti-Diaphanous 1 (Thermo #A300-077A) and anti-GAPDH (Invitrogen #AM4300) followed by appropriate HRP-conjugated secondary antibodies. The membrane was developed using Pierce ECL Western Blotting Substrate and imaged on an Amersham Imager 680.

## Results

### Case description

**Figure 1:**
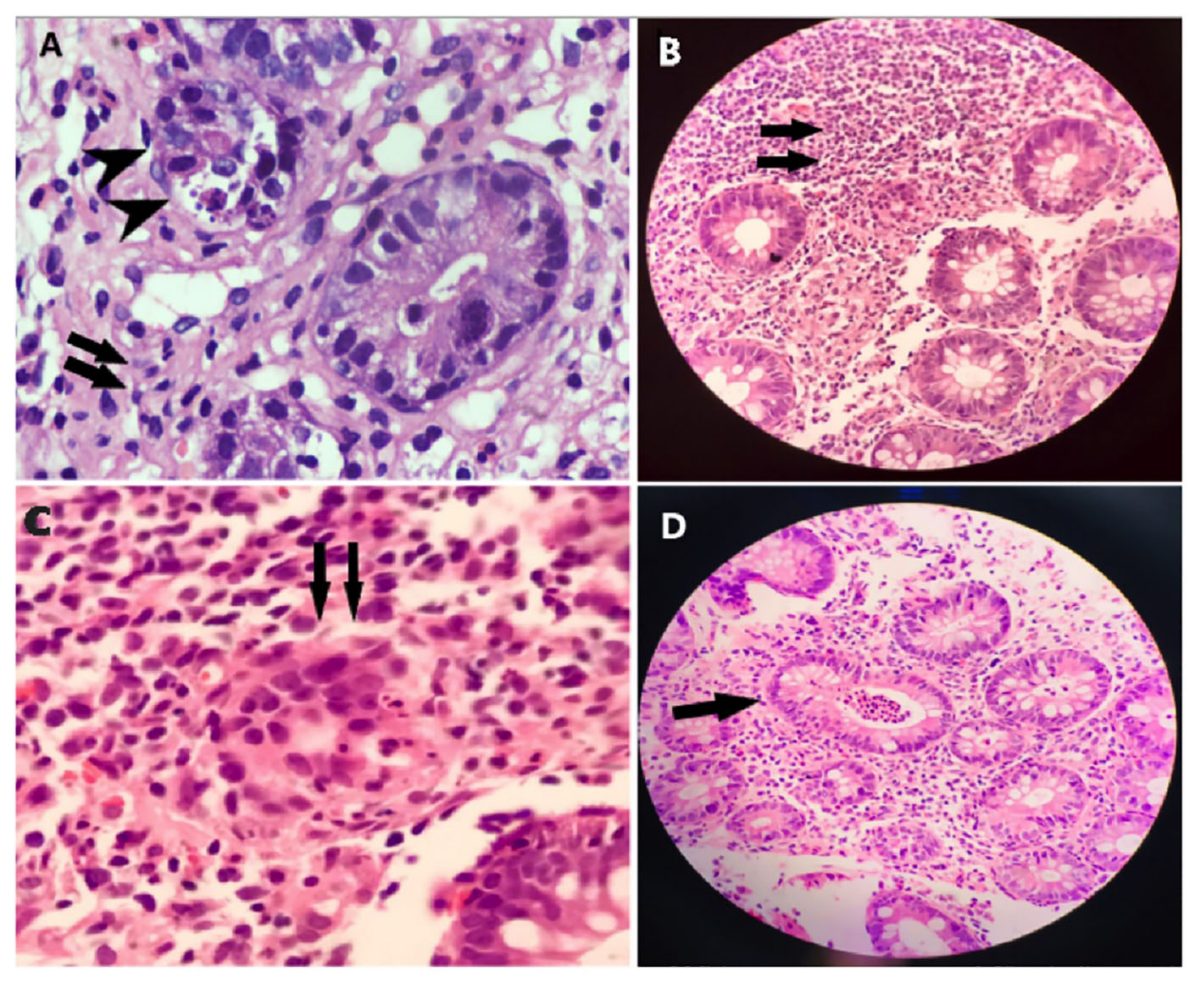
Histopathology of intestinal biopsy samples suggests inflammation and enteropathy. **(A)** Duodenal mucosal biopsy was performed and revealed sparse infiltration by lymphocytes, and eosinophils, indicated by arrows. **(B)** Sigmoid mucosal biopsy showed mild colitis, with lymphocytic and neutrophilic infiltration, indicated by arrows, in the lamina propria. **(C)** Apoptotic bodies, indicated by arrows, were noted in the crypts. **(D)** Apoptotic bodies, indicated by arrows, were noted in the crypt abscess.

**Figure 2:**
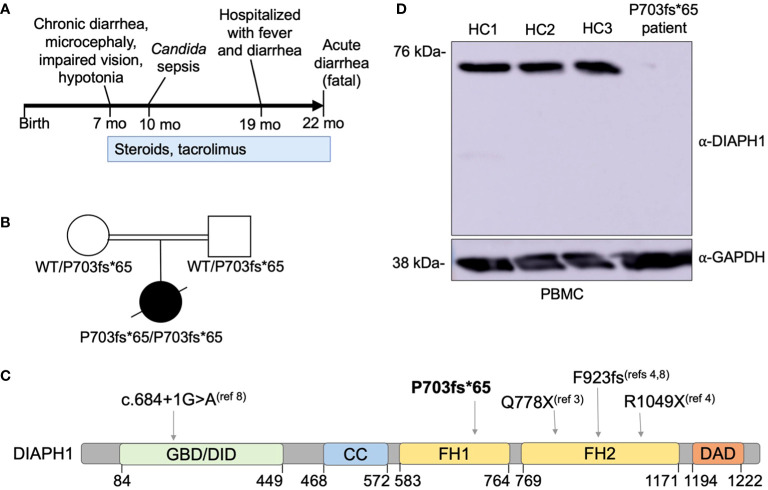
A novel *DIAPH1* mutation ablating protein expression in a patient with enteropathy, microcephaly and cortical blindness. **(A)** Schematic overview of the time course regarding symptom presentation and treatment. **(B)** Pedigree of the patient’s family. Double bar indicates parental consanguinity. A novel homozygous *DIAPH1* mutation c.2081delC, causing frameshift mutation P703fs*65 in the DIAPH1/mDia1 protein, was identified using trio-design whole exome sequencing. **(C)** Schematic of the DIAPH1 protein, showing the location of variants causing an autosomal recessive disease that includes both Seizures, Cortical blindness, and Microcephaly Syndrome (SCBMS) and variable immune deficiency and dysregulation. GBD denotes GTPase binding domain, DID denotes Dia inhibitory domain, CC denotes coiled-coil domain, FH1 denotes formin homology domain 1, FH2 denotes formin homology domain 2, DAD denotes Dia autoregulatory domain. Amino acid locations are indicated below. **(D)** Western blot using protein lysate prepared from peripheral blood mononuclear cells (PBMC) of three healthy controls (HC1-HC3) and the patient.

This case concerns a 7-month-old female patient who presented with chronic diarrhea, developmental delay and failure to thrive. On examination she was noted to have microcephaly (occipital frontal circumference 37cm), impaired vision due to cortical blindness, hypotonia and developmental delay. The patient had no history of seizures. She was born at term and had no history suggestive of birth asphyxia. At the time of presentation, infectious explanations for her severe diarrhea (~30 stools/day) were sought. CMV testing was negative and a stool BioFire GI Panel (BioMerieux) for 22 intestinal pathogens was also negative (**Supplementary Data**). Tests for tissue transglutaminase antibodies (TTGA) and anti-deamidated gliadin peptides (GDP) were negative. Endoscopy of the upper and lower GI tract was performed. Duodenal villi appeared blunted, and patchy nodularity was noted. Duodenal mucosal biopsy was performed and revealed sparse infiltration by lymphocytes and eosinophils (**Figure 1A**). A sigmoid mucosal biopsy showed mild colitis, with lymphocytic and neutrophilic infiltration in the lamina propria (**Figure 1B**). A note was also made of few apoptotic bodies in the crypts (**Figure 1C**) and crypt abscess (**Figure 1D**). These histopathology results, combined with negative results for various causes of infectious enteropathy, suggested very early onset inflammatory bowel disease. The patient was treated with steroids and tacrolimus, and her chronic diarrhea and enteropathy lessened but did not completely resolve. The patient had one episode of *Candida* sepsis at age 10 months. She was briefly hospitalized with fever and diarrhea (culture negative) at age 19 months. She had no history of lower respiratory tract infections, ear infections, or seizures. Unfortunately, the child expired at 22 months of age, when she presented with acute diarrhea with underlying chronic malnutrition (weight 5.3 kg) (**Figure 2A**). The patient’s parents had no history of similar symptoms and the patient had no siblings.

### Immunological and genetic investigations

Immunophenotyping by flow cytometry was performed when the patient was 11 months of age (**Table 1**) and revealed low lymphocyte count, and low total T cells attributable to a reduced CD4+ T cell compartment. Within both CD4+ and CD8+ T cells, naïve cells were markedly decreased and central memory cells were expanded, relative to the age-matched healthy control range. CD4-CD8- T cells were also present at an increased frequency, and IgE level was elevated, relative to the reference ranges (**Table 1**). In addition, recent thymic emigrants were very low to undetectable at <1 cell/µL. This patient’s clinical presentation and immunophenotyping results prompted a search for an underlying inborn error of immunity.

**Table 1:** Immunophenotyping results of the patient with age-matched reference ranges.

Measurement	Patient (aged 11 months)	Age-matched reference range
Absolute lymphocyte count	**1898/µL**	4000-10500/µL
CD3+ T cells	**42% (L)** **716/µL**	56-87%
CD4+ T cells	**13% (L)** **227/µL**	25-86%
Naïve CD45RA+CCR7+ (% of CD4)	**1.4% (L)** **3/µL**	77-96%
Central memory CD45RA-CCR7+ (% of CD4)	**51% (H)** **117/µL**	7-22%
Effector memory CD45RA-CCR7- (% of CD4)	**42% (H)** **97/µL**	0.008-4%
Terminally differentiated CD45RA+CCR7- (% of CD4)	2.3% 5/µL	0.0001-2.7%
CD8+ T cells	22% 374/µL	7-58%
Naïve CD45RA+CCR7+ (% of CD8)	**0.5% (L)** **2/µL**	16-100%
Central memory CD45RA-CCR7+ (% of CD8)	**29.9% (H)** **112/µL**	2-6%
Effector memory CD45RA-CCR7- (% of CD8)	23.4% 88/µL	1-100%
Terminally differentiated CD45RA+CCR7- (% of CD8)	42% 158/µL	4-92%
CD4-CD8- double negative T cells	**15% (H)** **110/µL**	0.4-2%
CD19+ B cells	4% 68/µL	3-77%
CD56+ NK cells	12% 204/µL	1-64%
IgG	622 mg/dl	172- 1069 mg/dl
IgE	**346 IU/mL (H)**	1.4-52 IU/mL
IgA	83 mg/dl	11- 106 mg/dl
IgM	85 mg/dl	41-173 mg/dl

Values in bold font indicate results outside the reference range; L, low; H, high.

Due to 3^rd^ degree parental consanguinity (**Figure 2B**), an autosomal recessive gene defect was suspected. Trio-design WES analysis revealed a single nucleotide deletion at Chr5:141573742, c.2081delC, causing frameshift mutation P703Hfs*65 in *DIAPH1*. This variant was not present in the gnomAD v3.1.1 database (2). The P703Hfs*65 mutation was homozygous in the patient and heterozygous in each parent (**Figure 2B**). This mutation is predicted to truncate DIAPH1 in the Lasso domain of the protein (**Figure 2C**), similar to the previously-reported Q778X mutation (3). Immunoblotting using patient PBMC showed no detectable DIAPH1 protein, in contrast to PBMC from three healthy controls (**Figure 2D**). Collectively, these data identify a novel mutation in DIAPH1 that causes severe monogenic disease in the biallelic state, and these findings also suggest that DIAPH1 deficiency may cause a wide variety of immunological phenotypes including severe inflammation.

## Discussion

Less than ten years ago, rare bi-allelic mutations in *DIAPH1* were shown to cause an autosomal recessive disease called Seizures, Cortical blindness, and Microcephaly Syndrome (SCBMS) (3). More recently, investigations of additional patients with bi-allelic *DIAPH1* mutations have shown that the phenotype of this rare monogenic disease features prominent and potentially fatal immune deficiency and dysregulation, in addition to the previously recognized SCBMS phenotypes. Including this report, *DIAPH1* biallelic mutations have now been identified in 17 patients from 9 families (3, 4, 8) (**Table 2**). The spectrum of phenotypes caused by DIAPH1 deficiency may expand further as additional cases come to light. The present case describes a child with the features of SCBMS but who also had prominent, early-onset diarrhea with autoimmune or inflammatory enteropathy. In this case, immune dysregulation was accompanied by mildly reduced total T cells, markedly reduced CD4+ T cells, and CD4+ and CD8+ T cell subsets heavily skewed toward memory cells (**Table 1**). These immunological phenotypes have been reported in some other cases of DIAPH1 deficiency (8), suggesting that these cellular phenotypes, in combination with clinical presentation, may be useful for the diagnosis of DIAPH1 deficiency. This patient also had a marked expansion of CD4-CD8- double negative T cells, a phenotype not previously reported in DIAPH1-deficient patients but present in LAT deficiency (6, 7). Unlike other reported cases of DIAPH1 deficiency, this child did not have seizures nor respiratory infections and had only one major infection with *Candida*. These results indicate that signs of immune dysregulation, such as inflammatory bowel disease, may be the most prominent symptom in infants with DIAPH1 deficiency. Since the microcephaly, visual impairment and developmental delay are present only postnatally, and vary in pace of progression, immune dysregulation may be the initial symptom in some patients.

**Table 2:** Clinical manifestations of DIAPH1 deficiency in patients reported to date, including this manuscript.

Phenotype	Prevalence in Q778X/Q778X patients (3) (Pakistan)	Prevalence in F923fs/F923fs patients (4, 8) (Oman)	Prevalence in R1049X/R1049X patients (4) (UAE)	Prevalence in c.684 + 1G>A/ c.684 + 1G>A patients (8) (Finland)	Present case P703fs/ P703fs (India)
Microcephaly	5/5	3/3	3/3	5/5	yes
Visual impairment	5/5	3/3	3/3	5/5	yes
Developmental delay	4/5 (youngest patient not assessed)	3/3	3/3	5/5	yes
Low height and weight for age	5/5	1/1	2/3 (not reported in youngest patient)	2/5	yes
Seizures	5/5	3/3	3/3	5/5	no
Lung phenotypes	2/5 (bronchiectasis requiring surgery; fatal lung infection)	2/3 (recurrent pulmonary infections)	1/3 (bronchiectasis, fatal lung infection)	3/5 (recurrent infections; bronchiolitis)	no
Other infections	None reported	2/3 (multiple including otitis media, *Candida*, mycobacteria, *Molluscum contagiosum*, EBV, VZV, HSV)	None reported	5/5 (multiple including otitis media, *Molluscum contagiosum*, *Candida*, *Staph hemolyticus*, *Strep pneumonia*, EBV, CMV, RSV, JCV)	*Candida* sepsis
Enteropathy and diarrhea	None reported	None reported	None reported	1/5	yes

While microcephaly, visual impairment, and developmental delay are common to all patients in the literature, immunological and infectious phenotypes show inter-individual variability.

All DIAPH1-deficient patients reported to date have homozygous mutations that result in aberrant splicing or premature truncation of the DIAPH1 protein, and the majority of these mutations have been experimentally shown to abolish DIAPH1 expression (3, 4, 8). Further research is needed to understand the functional significance of other DIAPH1 mutations, which may preserve protein expression but impair its function. Interestingly, a heterozygous premature truncation of DIAPH1 was first reported to cause fully-penetrant autosomal dominant sensorineural deafness and macrothrombocytopenia (also called DFNA1) in a large Costa Rican kindred (13). Other kindreds with DIAPH1 gain-of-function mutations have been described, and the molecular mechanism of disease in this setting is attributed to a mutation affecting the autoregulatory domain (14, 15). The fact that different DIAPH1 truncations can have vastly different biological consequences suggests there is still much to learn about the role of this and other formins in human physiology, and immunology in particular.

Diagnosis of all DIAPH1-deficient patients reported to date has relied on genetic testing (3, 4, 8). When reported, the prenatal and perinatal histories of these patients are characterized by normal prenatal ultrasounds, normal delivery, and normal growth parameters at birth (3, 4, 8). These results indicate that standard prenatal screening (without genetic testing) will not be sufficient to detect DIAPH1 deficiency. In some families, known parental consanguinity and the presence of symptoms in older children may prompt genetic counselling and/or prenatal genetic testing. However, the enrichment of the DIAPH1 splicing variant c.684 + 1G>A in Finland (8) presents unique challenges, as most children in the Finnish families did not have affected siblings, and none were born to consanguineous parents.

Treatment of DIAPH1-deficient patients is very challenging. Several reports indicate that seizures in these patients are refractory to anti-epileptic therapies (3, 4, 8). Several DIAPH1-deficient patients have had uncontrolled EBV infections. One patient had EBV viremia at age 19, and subsequently developed diffuse large B-cell lymphoma (8). Another patient died of EBV-positive B-cell lymphoma at age 3 years (8). These findings underscore the importance of monitoring DIAPH1-deficient patients for EBV-related malignancy. Fewer than 20 patients with autosomal recessive DIAPH1 deficiency have been reported (3, 4, 8). As the number of patients diagnosed with this monogenic disease grows, it is likely that the spectrum of clinical phenotypes, and especially immunological phenotypes, will also expand. These findings, together with recent publications, suggest that DIAPH1 deficiency should be re-defined as a monogenic cause of both SCBMS and primary immunodeficiency with immune dysregulation.

## Data availability statement

The original contributions presented in the study are included in the article/**Supplementary Material**. Variant call files for WES from the patient and parents included in this study (patient BHATT005-1; mother BHATT005-2; father BHATT005-3) are available via the Open Science Framework at https://osf.io/k2a5h/. Further inquiries can be directed to the corresponding author.

## Ethics statement

This study was performed in line with the principles of the Declaration of Helsinki and was approved by the Institution Review Board at Vanderbilt University Medical Center. Written informed consent was obtained for adult participants and parental consent was obtained for the patient. The authors affirm that human research participants provided informed consent for publication of the results generated in this study.

## Author contributions

SB: Formal analysis, Investigation, Writing – review & editing. SR: Formal analysis, Investigation, Writing – review & editing. RK: Formal analysis, Investigation, Writing – review & editing. JC: Formal analysis, Visualization, Writing – review & editing. JM: Data curation, Funding acquisition, Supervision, Writing – original draft, Writing – review & editing.
