# Supplementary material for: Immune dysregulation due to bi-allelic mutation of the actin remodeling protein DIAPH1
Source: Front Immunol. 2024 Jul 15;15:1406781. doi: 10.3389/fimmu.2024.1406781 (PMC11284534; doi:10.3389/fimmu.2024.1406781)
Supplement: Supplementary file 1 [file DataSheet_1.docx]

Supplementary data for:

Immune dysregulation due to bi-allelic mutation of the

actin remodeling protein DIAPH1

**Sagar Bhattad^1^, Somashekara H. Ramakrishna^2^, Ratan Kumar^3^, Joseph M. Choi^4^, Janet G. Markle^4,5,6,*^**

^1^Division of Pediatric Immunology and Rheumatology, Department of Pediatrics, Aster CMI Hospital, Bengaluru, India

^2^Gleneagles Global Hospitals, Bengaluru, India

^3^Department of Pediatrics, Narayana Health City, Bengaluru, India

^4^Division of Molecular Pathogenesis, Department of Pathology Microbiology and Immunology, Vanderbilt University Medical Center, Nashville TN, USA

^5^Vanderbilt Genetics Institute, Division of Genetic Medicine, Department of Medicine, Vanderbilt University Medical Center, Nashville TN, USA

^6^Vanderbilt Center for Immunobiology and Vanderbilt Institute for Infection, Immunology and Inflammation, Nashville TN, USA

*** Correspondence:**Janet Markle
janet.markle@vumc.org

Keywords: pediatrics, medical genetics, autoimmunity, enteropathy, inborn errors of immunity, inflammatory bowel disease

Supplementary Table 1: Results of clinical testing for infectious etiologies using BioFire FilmArray GI Panel

| **Bacteria** | |
| --- | --- |
| Not detected | Campylobacter |
| Not detected | Clostridium difficile toxin A/B |
| Not detected | Plesiomonas shigelloides |
| Not detected | Salmonella |
| Not detected | Vibro |
| Not detected | Vibrio cholerae |
| Not detected | Yersinia enterocolitis |
| **Diarrheagenic E. coli / Shigella** | |
| Not detected | Enteroaggregative E. coli (EAEC) |
| Not detected | Enteropathogenic E. coli (EPEC) |
| Not detected | Enterotoxigenic E. coli (ETEC) |
| Not detected | Shiga-like toxin-producing E. coli (STEC) |
| Not detected | Shigella/Enteroinvasive E. coli (EIEC) |
| N/A | E. coli O157 |
| **Parasites** | |
| Not detected | Cryptosporidium |
| Not detected | Cyclospora cayetanensis |
| Not detected | Entamoeba histolytics |
| Not detected | Giardia lamblia |
| **Viruses** | |
| Not detected | Adenovirus F 40/41 |
| Not detected | Astrovirus |
| Not detected | Norovirus GI/GII |
| Not detected | Rotavirus A |
| Not detected | Sapovirus |
